# The keys to success: initial findings from the Hand Hygiene Australia (HHA) program review

**DOI:** 10.1186/2047-2994-4-S1-P144

**Published:** 2015-06-16

**Authors:** K Ryan, S Havers, K Olsen, A Stewardson, M Cruickshank, ML Grayson

**Affiliations:** 1 Hand Hygiene Australia, Australia; 2 Australian Commission on Safety and Quality in Health Care, Australia

## Introduction

The Australian Commission on Safety and Quality in Health Care engaged HHA to implement the National Hand Hygiene Initiative (NHHI) in 2008. The NHHI is based on the World Health Organisation clean care is safer care program. In 2014 HHA was asked to review hospital hand hygiene (HH) programs to evaluate their alignment with the NHHI.

## Objectives

To review the validity of HHC data, and to identify innovations and key components of a successful HH program.

## Methods

HHA selected healthcare facilities across each Australian state and territory based on size (>300 beds) and high reported HHC. Program Reviews (PR) were conducted by the national HHA team, and consisted of a structured interview with hospital staff responsible for HH promotion, and side-by-side auditing. The interview covered the 5 key components of the WHO multimodal strategy: system change, education and training, monitoring and performance feedback, reminders in the workplace, and institutional safety climate.

## Results

22 healthcare organisations were visited (median HHC 81%, range 71-90.6%). All had HH products at the point of care. The higher performing facilities included HH in all teaching activities, monitored HH online learning package completion rates, and conducted targeted education dependent on audit results. All sites reported regular auditing conducted by ward-based auditors in 19/22 sites. 13 (59%) sites audited all wards. There was validation of data and regular and timely performance feedback to all stakeholders at all sites. The annual auditor validation process was monitored in 11/22 sites, with 12 sites running auditor refresher training as required. Minor auditing inconsistencies were detected during side-by-side auditing at 6 sites. Of those, 5/6 did not monitor the annual auditor validation, nor run refresher sessions. The PRs identified several local innovations and reinforced the importance of local ownership to achieve exceptional results.

## Conclusion

All sites visited were well aligned with the NHHI. However, auditing of all wards and annual auditor validation were identified as areas for improvement. Overall, these PRs support the validity of data submitted as a part of the NHHI, and have provided new ideas about how to implement HH improvement.

## Disclosure of interest

None declared.

